# Smoking, Gender, and Overweight Are Important Influencing Factors on Monocytic HLA-DR before and after Major Cancer Surgery

**DOI:** 10.1155/2017/5216562

**Published:** 2017-08-08

**Authors:** Gunnar Lachmann, Clarissa von Haefen, Johannes Kurth, Fatima Yuerek, Klaus-Dieter Wernecke, Claudia Spies

**Affiliations:** ^1^Department of Anesthesiology and Operative Intensive Care Medicine, Charité - Universitätsmedizin Berlin, Campus Charité Mitte and Campus Virchow-Klinikum, Berlin, Germany; ^2^Sostana GmbH, Berlin, Germany

## Abstract

**Background:**

Monocytic human leukocyte antigen D related (mHLA-DR) is essential for antigen-presentation. Downregulation of mHLA-DR emerged as a general biomarker of impaired immunity seen in patients with sepsis and pneumonia and after major surgery. Influencing factors of mHLA-DR such as age, overweight, diabetes, smoking, and gender remain unclear.

**Methods:**

We analyzed 20 patients after esophageal or pancreatic resection of a prospective, randomized, placebo-controlled, double-blind trial (placebo group). mHLA-DR was determined from day of surgery* (od)* until postoperative day* (pod) 5*. Statistical analyses were performed using multivariate generalized estimating equation analyses (GEE), nonparametric multivariate analysis of longitudinal data, and univariate post hoc nonparametric Mann–Whitney tests.

**Results:**

In GEE, smoking and gender were confirmed as significant influencing factors over time. Univariate analyses of mHLA-DR between smokers and nonsmokers showed lower preoperative levels (*p* = 0.010) and a trend towards lower levels on* pod5* (*p* = 0.056) in smokers. Lower mHLA-DR was seen in men on* pod3* (*p* = 0.038) and on* pod5* (*p* = 0.026). Overweight patients (BMI > 25 kg/m^2^) had lower levels of mHLA-DR on* pod3* (*p* = 0.039) and* pod4* (*p* = 0.047).

**Conclusion:**

Smoking is an important influencing factor on pre- and postoperative immune function while postoperative immune function was influenced by gender and overweight. Clinical trial registered with ISRCTN27114642.

## 1. Background

Antigen-presenting cells, that is, macrophages, dendritic cells, and activated natural killer cells, express human leukocyte antigen D related (HLA-DR) on their surface which is crucial for immunologic competence [1, 2]. Immunogenic peptides processed from ingested pathogens, for example, bacterial proteins, are presented to T cells by HLA-DR. Specific T cell antigen receptors recognize the HLA-DR-antigen complex and subsequently activate the T cells [3]. Thus, the expression of HLA-DR reflects the functional state of antigen-presenting cells.

Downregulation of HLA-DR expression on monocytes (mHLA-DR) emerged as a general biomarker of impaired immunity up to immune suppression which can predict outcome [1, 4, 5]. In the state of reduced mHLA-DR expression, the function of monocytes and T cell activation is severely restricted [6] which results in up to five times higher risk for infections and septic complications [7–9]. A reduced mHLA-DR expression is associated with sepsis, pneumonia, and surgical site infections [9–11] and furthermore seen in patients after thoracic [12, 13] and major visceral surgery [1, 3, 14], in trauma patients [15, 16] and patients with burn injury [17].

We and others used mHLA-DR expression to stratify immune compromised patients after surgery [18, 19] and during severe sepsis [20]. In both settings, mHLA-DR could be increased through administration of GM-CSF restoring immune function and improving outcome. However, reasons for a decreased mHLA-DR expression frequently seen after major surgery and during sepsis are not clearly understood. Surgical stress and tissue damage resulting in an inflammatory state are proposed to downregulate mHLA-DR through cortisol and interleukin- (IL-) 10 [21, 22].

Various conditions can alter immune function generally and particularly after surgery. Ageing [23, 24], obesity [25], diabetes [26, 27], gender [1], and smoking [28, 29] have an influence on the immune function, immune cells, and cytokines. Smoking is suggested to trigger the development of rheumatoid arthritis or multiple sclerosis due to interactions between subtypes of HLA-DR alleles [30–32]. Furthermore, smokers showed a decreased T cell function and a higher rate of HLA-DR positive T cells, that is, a higher activation [33]. Ono et al. found lower levels of mHLA-DR in men compared to women on the first day after gastrectomy [1]. However, the influence of age, BMI, diabetes, and smoking on pre- and postoperative mHLA-DR and immune function is unclear. Thus, we analyzed mHLA-DR after major cancer surgery regarding influencing factors such as age, overweight, diabetes, smoking, gender, and surgical time.

## 2. Patients and Methods

### 2.1. Study Participants and Design

**Figure 1:**
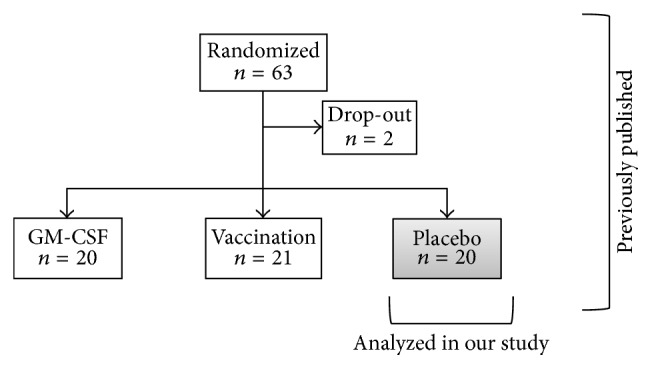
Consort diagram.

This retrospective subgroup analysis refers to a previously published study of our research group [18] which studied mHLA-DR in 20 out of 61 immune suppressed patients (mHLA-DR levels below 10,000 mAb per cell on* pod1*) from* od *until* pod5 *after elective esophageal or pancreatic resections since these subgroup patients were not treated with study medication (Figure 1). All patients received guideline-based anesthesiological and surgical treatment according to our standard operating procedures [34].

### 2.2. Measurement of mHLA-DR, Leukocytes, and C-Reactive Protein

Blood samples were taken from* od* until* pod5* and intraoperative parameters (surgical time, blood glucose and lactate levels, and blood pressure) were documented. mHLA-DR, leukocytes, and C-reactive protein (CRP) were measured in all patients from* od* until* pod5* in cooperation with the Institute of Medical Immunology and Berlin-Brandenburg Center for Regenerative Therapies (BCRT), Charité - Universitätsmedizin Berlin, Berlin, Germany. mHLA-DR was measured using a highly standardized quantitative assay as described earlier [8]. Plasma levels of CRP were measured by an immunoturbidimetric assay (Roche Diagnostics, Mannheim, Germany) whereas white blood cell analyses were performed on a standard hematology analyzer (Sysmex GmbH, Norderstedt, Germany).

### 2.3. Clinical Outcome Parameters

During the follow-up period until* pod9*, we determined infections according to the standards of the CDC and ATS criteria for pneumonia [35, 36], incidence of delirium using Delirium Detection Score (DDS, [37]) with a DDS > 3, and hospital and ICU stay.

### 2.4. Statistical Analysis

Data were expressed according to their scaling as arithmetic mean ± standard deviation (SD), median [25%, 75% quartiles], or frequencies [%], respectively. After exploratory data analysis, all tests were accomplished by means of nonparametric exact statistical tests. Basic patient characteristics were evaluated for group differences using the Mann–Whitney tests for continuous variables and the Fisher exact test for categorical variables. To detect influences on mHLA-DR, we used multivariate generalized estimating equation (GEE) over the six time points with mHLA-DR as dependable variable and gender, smoking, diabetes, BMI, age, and surgical time as independent variables. Significant variables in GEE were taken as risk factors for mHLA-DR in a multivariate nonparametric analysis of longitudinal data in a two-factorial design and judged for corresponding impact. This analysis tests for three hypotheses, namely, differences in risk factors, systematic changes over time, and interactions between differences and time. After such global testing, univariate tests were further carried out as post hoc analyses to detect specific differences with respect to those groups for fixed times (exact Mann–Whitney tests). A two-tailed *p* value < 0.05 was considered statistically significant. All tests were conducted in the area of exploratory data analysis. Therefore, no adjustments for multiple testing have been made. The calculations were performed with IBM© SPSS© Statistics, Version 23 and SAS, Version 9.1, Copyright© by SAS Institute, Inc., Cary, NC, USA.

## 3. Results

### 3.1. Study Population

All analyses were performed in 20 patients of the placebo group from* od *until* pod5*. mHLA-DR, leukocytes, and CRP were measured in all patients.

### 3.2. Multivariate Analyses (GEE) of Influencing Factors on mHLA-DR

Multivariate GEE was conducted in 20 patients from* od *until* pod5 *using mHLA-DR as responder and diabetes, BMI (dichotomized as overweight with ≤25 versus >25 kg/m^2^), smoking, gender, surgical time, and age as influencing factors. Smoking (*p* = 0.002) and gender (*p* = 0.026) were confirmed as influencing factors over time. Therefore, smoking and gender were taken as risk factors in multivariate longitudinal analyses together with overweight for clinical reasons.

### 3.3. Influence of Smoking on mHLA-DR

**Figure 2:**
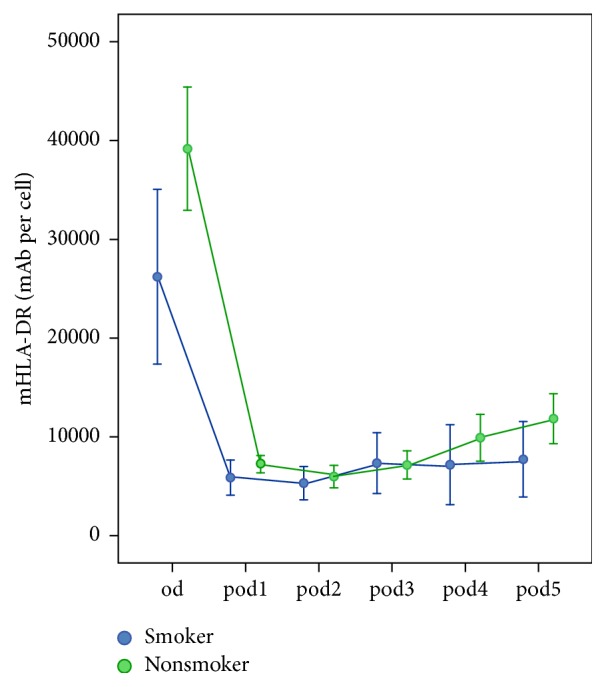
Monocytic HLA-DR (mHLA-DR) immediately before surgery* (od)* until day 5 after surgery* (pod5)* between smokers and nonsmokers (error bars with 95% confidence interval). mHLA-DR was lower in smokers on* od* (*p* = 0.010),* pod4 *(*p* = 0.098), and* pod5 *(*p* = 0.056).

8 out of the 20 patients were active smokers. Differences in basic patient characteristics did not differ between smokers and nonsmokers. The longitudinal analysis revealed no significant risk factor differences (*p* = 0.142) but resulted in significant changes over time (*p* < 0.001) and interactions (*p* = 0.038), which showed increasing differences over time. In univariate analyses, preoperative mHLA-DR was significantly lower in smokers (*p* = 0.010; Figure 2) whereas a trend towards lower levels was seen on* pod4* (*p* = 0.098) and* pod5* (*p* = 0.056) in smokers. Leukocytes and CRP did not differ from* od* until* pod5 *except for a trend towards higher CRP on* pod1* (*p* = 0.053) in smokers. Furthermore, smokers revealed a longer ICU stay (*p* = 0.047). Differences are shown in Table 1.

### 3.4. Influence of Overweight on mHLA-DR

**Figure 3:**
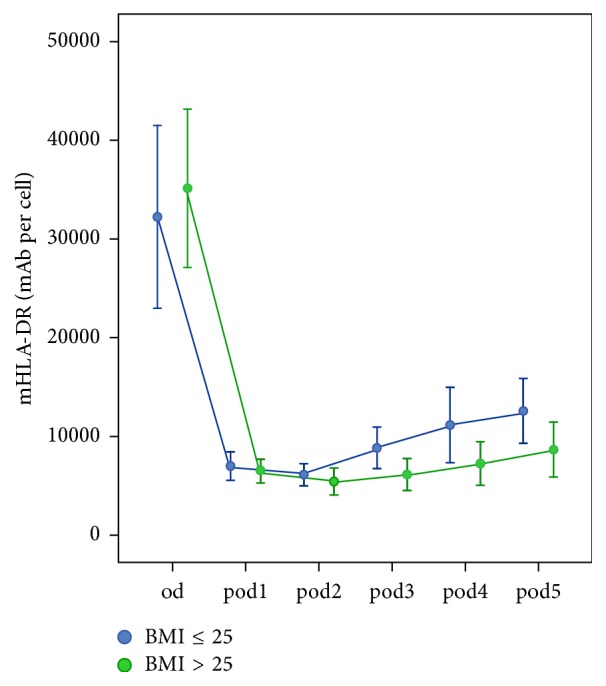
Monocytic HLA-DR (mHLA-DR) immediately before surgery* (od)* until day 5 after surgery* (pod5)* between normal weight and overweight patients (error bars with 95% confidence interval). Lower levels of mHLA-DR were seen on* pod3 *(*p* = 0.039) and on* pod4 *(*p* = 0.047) in overweight patients.

Groups were divided into normal weight (BMI ≤ 25 kg/m^2^) and overweight (BMI > 25 kg/m^2^). 12 out of 20 patients were overweight. No differences in basic patient characteristics occurred. The longitudinal analysis revealed no significant risk factor differences (*p* = 0.079) but resulted in significant changes over time (*p* < 0.001) and interactions (*p* = 0.011), which showed increasing differences over time. In univariate analyses, mHLA-DR was lower in overweight patients on* pod3 *(*p* = 0.039) and on* pod4 *(*p* = 0.047; Figure 3). Leukocytes, CRP, and outcome parameters did not differ between normal weight and overweight patients. Differences are shown in Table 2.

### 3.5. Influence of Gender on mHLA-DR

**Figure 4:**
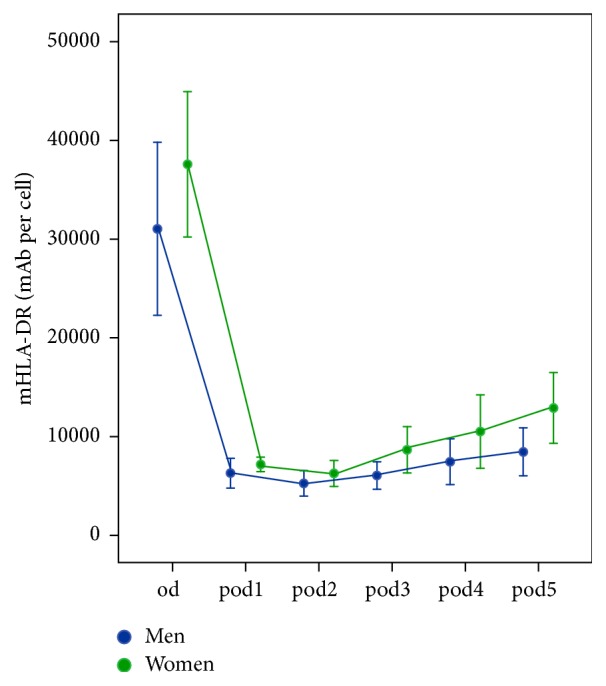
Monocytic HLA-DR (mHLA-DR) immediately before surgery* (od)* until day 5 after surgery* (pod5)* between men and women (error bars with 95% confidence interval). mHLA-DR was lower in men on* pod3* (*p* = 0.038) and on* pod5* (*p* = 0.026).

11 out of 20 patients were male. Differences in basic patient characteristics occurred in BMI. The longitudinal analysis revealed no significant risk factor differences (*p* = 0.067) but resulted in significant changes over time (*p* < 0.001). In univariate analyses, mHLA-DR was lower in men on* pod3 *(*p* = 0.038) and on* pod5 *(*p* = 0.026; Figure 4). Leukocytes and outcome parameters did not differ between the groups. CRP was higher in men on* pod1 *(*p* = 0.019). Differences are shown in Table 3.

## 4. Discussion

The major finding of this subanalysis is that pre- and postoperative mHLA-DR were influenced by smoking, gender, and overweight. Smokers revealed an attenuated pre- and postoperative immune function, whereas recovery of mHLA-DR was better in normal weight patients, women, and nonsmokers. This is an important finding, particularly with regard to the impact of smoking on pre- and postoperative function on the immune system. To the best of our knowledge, no other study has investigated influencing factors on postoperative mHLA-DR up to date.

HLA-DR is a key molecule on antigen-presenting cells and a marker of impaired immunity and immune suppression, for example, during sepsis and after surgery [1, 4, 5]. We found lower values of mHLA-DR preoperatively in smokers as well as higher levels of CRP and a delayed recovery of mHLA-DR after surgery. Smoking might therefore have direct influences on pre- and postoperative immune function, a generally lower immunity, and delayed recovery of immune suppression after surgery. Most likely, these alterations resulted in outcome differences seen in a prolonged ICU stay in smokers. Shiels et al. found a decrease of inflammatory markers and an increase in CRP in long-term smokers [29] whereas Hiemstra et al. could show that inflammation is increased and host defence against infections is decreased by cigarette smoke [38]. In accordance with Hiemstra et al., we observed an increased inflammation by CRP and a decreased host immunity by mHLA-DR. Dogan et al. conclude that regular smoking correlates with alterations in peripheral mononuclear cells interfering inflammatory and immune function pathways [28]. In previous studies, smoking showed significant interactions with subtypes of HLA-DR alleles and therefore increased the risk of developing rheumatoid arthritis or multiple sclerosis [30–32]. Unfortunately, our study was not designed to analyze these interactions but most likely smoking influences not only subtypes of HLA-DR alleles, but also mHLA-DR expression. Our analyses show that smoking is not only related to postoperative complications such as cardiovascular [39], respiratory [40], and wound complications [41], but also related to preoperative immunological complications that might influence or induce postoperative complications. We hypothesize that regular smoking has long-term effects on immunity through directly affecting mHLA-DR. After surgery, mHLA-DR remains impaired resulting in a delayed recovery which increases the risk of postoperative complications [9]. Further studies are warranted to evaluate potential pathological mechanisms.

Additionally, we found a delayed recovery of mHLA-DR in overweight patients after surgery. Likewise diabetes, obesity generally constitutes a chronic inflammatory state, particularly elevated leukocyte counts, activity of granulocyte phagocytosis and oxidative burst, and increased levels of TNF-*α* and IL-6 [23, 25, 26]. Obesity is also an important risk factor for increased mortality up to 20% and a shortened life expectancy up to 2 to 4 years [42, 43]. In various surgical procedures, overweight or obesity can lead to an increased risk of postoperative infections [44–47]. We have not seen increased CRP or leukocytes in overweight patients but an increased risk for postoperative complications by a delayed recovery of mHLA-DR [9]. In summary, overweight patients had a postoperatively impaired immunity and thus most likely an increased risk for postoperative complications.

In GEE, we found differences in postoperative mHLA-DR between men and women over time and lower postoperative levels in men in univariate analyses. Gender generally constitutes an unmodifiable risk factor for postoperative complications [48]. Ono et al. investigated postoperative mHLA-DR and also found lower levels in men on day one after gastrectomy but did not study further courses as we did [1]. In clinical studies, male patients showed a 2 times higher risk for postoperative infections and a 1.6 times higher rate of pneumonia [49, 50] whereas women with nosocomial infections are at higher lethality up to 2 times [51–53]. Additionally, women showed better outcomes after sepsis [54, 55].

Age and surgical time are also described as influencing factors on postoperative immune function. Ono et al. found an increased activation of monocytes and raised levels of proinflammatory cytokines in elderly patients after surgery [24]. Sutherland et al. demonstrated a reduced immune response in elderly patients after surgery [56]. Surgical time contributes also as a risk factor for postoperative infections [57]. However, we did not see any influence of surgical time or age on mHLA-DR.

Limitations of these analyses are first a small sample size of 20 patients and therefore, no immune cells and cytokines except for leukocytes and CRP were analyzed. Though significant differences occurred, a higher sample size could have shown more reliable results. Furthermore, we analyzed mHLA-DR of postoperative immune suppressed patients with a threshold level for mHLA-DR of ≤10,000 mAb/cell. Hence, the influence on mHLA-DR in less suppressed or unsuppressed patients remains unclear.

## 5. Conclusion

Smokers revealed a preoperative impaired mHLA-DR expression and a delayed recovery after surgery. Overweight patients as well as men showed a delayed recovery of mHLA-DR after surgery. This is an important finding, particularly with regard to the impact of smoking on pre- and postoperative function on the immune system. Smoking and overweight are therefore risk factors for an impaired immunity after surgery and an increased risk for postoperative complications.

## Figures and Tables

**Table 1:** Basic patient characteristics and outcome parameters between smokers and nonsmokers from *od *until *pod5*.

	Smokers (*n* = 8)	Nonsmokers (*n* = 12)	*p* value
Age [years]	64 (56–69)	66 (55–75)	*0.521*
BMI [kg/m^2^]	25.9 (20.8–28.6)	25.4 (24.3–27.8)	*0.792*
Gender [m/f]	5/3	6/6	*0.670*
ASA score II/III [*n*]	3/5	9/3	*0.167*
Diabetes in history [no/yes]	7/1	9/3	*0.619*
Surgical time [min]	323 (305–405)	296 (231–367)	*0.305*
Periop. blood glucose [mg/dL]	124 (120–138)	131 (119–143)	*0.624*
Periop. blood lactate [mmol/L] (max.)	1.2 (0.9–1.3)	0.9 (0.8–1.1)	*0.057*
Periop. systolic blood pressure [mmHg]	122 (109–137)	114 (112–116)	*0.270*
CRP on *pod1 *[mg/dL]	8.3 (7.6–9.3)	12.2 (8.3–16.9)	*0.053*
ICU stay [d]	5.4 (3.0–8.9)	3.5 (1.2–4.5)	**0.047**
Hospital stay [d]	19.0 (12.3–36.0)	15.4 (12.3–18.8)	*0.384*
Delirium [no/yes]	3/5	6/6	*0.670*
Infection [no/yes]	2/6	7/5	*0.197*

**Table 2:** Basic patient characteristics and outcome parameters between normal (BMI ≤ 25 kg/m^2^) and overweight patients (BMI > 25 kg/m^2^).

	Normal weight (*n* = 8)	Overweight (*n* = 12)	*p* value
Age [years]	66 (61–72)	61 (54–74)	*0.384*
Gender [m/f]	3/5	8/4	*0.362*
ASA score II/III [*n*]	5/3	7/5	*1.000*
Smokers/nonsmokers [*n*]	3/5	5/7	*1.000*
Diabetes in history [no/yes]	6/2	10/2	*1.000*
Surgical time [min]	294 (231–388)	332 (287–383)	*0.343*
Periop. blood glucose [mg/dL]	128 (119–145)	127 (120–138)	*0.678*
Periop. blood lactate [mmol/L] (max.)	1.0 (0.8–1.2)	1.1 (0.8–1.2)	*0.792*
Periop. syst. blood pressure [mmHg]	115 (111–123)	115 (113–119)	*1.000*
ICU stay [d]	4.0 (0.9–5.6)	3.9 (2.9–4.9)	*0.678*
Hospital stay [d]	13.5 (11.1–29.4)	16.3 (13.9–22.2)	*0.427*
Delirium [no/yes]	4/4	5/7	*1.000*
Infection [no/yes]	5/3	4/8	*0.362*

**Table 3:** Basic patient characteristics and outcome parameters between men and women.

	Men (*n* = 11)	Women (*n* = 9)	*p* value
Age [years]	60 (54–67)	69 (60–76)	*0.067*
BMI [kg/m^2^]	27.2 (24.5–28.9)	24.3 (21.0–25.7)	**0.046**
Smokers/nonsmokers [*n*]	5/6	3/6	*0.670*
ASA score II/III [*n*]	9/2	3/6	*0.065*
Diabetes in history [no/yes]	9/2	7/2	*1.000*
Surgical time [min]	315 (281–385)	315 (235–394)	*0.766*
Periop. blood glucose [mg/dL]	131 (123–141)	123 (110–138)	*0.331*
Periop. blood lactate [mmol/L] (max.)	1.7 (1.1–2.3)	1.3 (1.1–1.7)	*0.503*
Periop. systolic blood pressure [mmHg]	116 (113–123)	113 (111–118)	*0.331*
CRP on *pod1 *[mg/dL]	10.6 (8.8–14.9)	7.7 (6.4–8.7)	**0.019**
ICU stay [d]	4.0 (2.9–4.9)	3.8 (0.9–5.3)	*0.456*
Hospital stay [d]	15.0 (12.0–23.0)	15.9 (12.4–25.8)	*1.000*
Delirium [no/yes]	4/7	5/4	*0.653*
Infection [no/yes]	4/7	5/4	*0.653*
